# Engaging distributed cortical and cerebellar networks through motor execution, observation, and imagery

**DOI:** 10.3389/fnsys.2023.1165307

**Published:** 2023-04-11

**Authors:** Julia U. Henschke, Janelle M. P. Pakan

**Affiliations:** ^1^Institute of Cognitive Neurology and Dementia Research, Otto-von-Guericke-University Magdeburg, Magdeburg, Germany; ^2^German Center for Neurodegenerative Diseases, Magdeburg, Germany; ^3^Center for Behavioral Brain Sciences, Universitätsplatz, Magdeburg, Germany

**Keywords:** motor imagery, cerebrocerebellar, neuroimaging, motor network, action observation, motor execution, distributed brain network, cerebellar function

## Abstract

When we interact with the environment around us, we are sometimes active participants, making directed physical motor movements and other times only mentally engaging with our environment, taking in sensory information and internally planning our next move without directed physical movement. Traditionally, cortical motor regions and key subcortical structures such as the cerebellum have been tightly linked to motor initiation, coordination, and directed motor behavior. However, recent neuroimaging studies have noted the activation of the cerebellum and wider cortical networks specifically during various forms of motor processing, including the observations of actions and mental rehearsal of movements through motor imagery. This phenomenon of cognitive engagement of traditional motor networks raises the question of how these brain regions are involved in the initiation of movement without physical motor output. Here, we will review evidence for distributed brain network activation during motor execution, observation, and imagery in human neuroimaging studies as well as the potential for cerebellar involvement specifically in motor-related cognition. Converging evidence suggests that a common global brain network is involved in both movement execution and motor observation or imagery, with specific task-dependent shifts in these global activation patterns. We will further discuss underlying cross-species anatomical support for these cognitive motor-related functions as well as the role of cerebrocerebellar communication during action observation and motor imagery.

## Introduction

Interacting with the environment around us results in an incoming flow of dynamic sensory information that is used to guide ongoing motor behaviors. However, the dynamic nature of our environment demands that our patterns of motor behavior must remain flexible and diverse. When navigating from point A to B we may step with our right leg on the first trip, next time we may initiate movement with our left leg because our weight is distributed differently, on the next trip we may rotate and walk backward for a few steps if someone behind us begins to speak. This constant adaptation of our motor movements in relation to incoming sensory input from the environment means that motor control must be inherently flexible, both planning and generating efferent motor commands based on the continuous interaction of motor and perceptual processes (Kawato, 1999; Wolpert and Ghahramani, 2000). Further, we are sometimes actively executing motor patterns and other times only covertly engaging with our surroundings-taking in sensory information, observing the movement of others, or mentally rehearsing without physical engagement. Observing the movements of others may be crucial for establishing successful social interactions (Rozzi and Fogassi, 2017) and both action observation and motor imagery can lead to improved motor learning (Chye et al., 2022). Imagining one’s own motor movements, also known as motor imagery, refers to the mental simulation of performing a physical action (Decety and Ingvar, 1990; Jeannerod, 2001). Fundamentally, motor imagery is a neural process of mental rehearsal of movement, including motor cognition but without any motor output. The very existence of this mental capacity immediately begs the question of how our brain subserves both the execution of motor movements and the imagining of those same motor actions.

In this review we explore the distributed brain networks that are involved in motor execution, observation, and imagery. In particular, we identify major brain regions that play a vital role in both direct motor control and indirect motor cognition. We explore the evidence for common distributed brain networks, largely involving motor, pre-motor, and parietal cortex, as well as subcortical structures, such as the cerebellum. These major brain areas are individually known to play well established roles in coordinated sensorimotor integration and motor execution, but are also connected by one of the largest projection tracks in the brain, the cortico-ponto-cerebellar pathway, and together they form important functional communication networks (Apps and Watson, 2013; Palesi et al., 2017; McAfee et al., 2022). A series of recent meta-analyses of functional neuroimaging data suggests that these shared distributed networks play a unique role in the execution, observation, and mental rehearsal of motor movements (Grèzes and Decety, 2000; Caspers et al., 2010; Molenberghs et al., 2012; Hétu et al., 2013; Hardwick et al., 2018), however, key functional specializations also differentiate activation across these conditions. First, we discuss the structural and functional implications of the extensive connections between these distributed brain networks and their potential coordinated role in motor execution, observation, and imagery. We then consider the challenges and limitations of investigating motor imagery on the level of neural circuits and discuss key future perspectives.

## Structure and function of distributed brain networks for motor processing

The generation, execution and ongoing adaptation of movements is fundamental to our daily lives and wellbeing. While uncovering fundamental principles of motor control has been a central focus of neuroscience research for centuries, many questions remain regarding the precise sequence of events that lead from thought to movement. Perhaps the most advanced understanding exists at the level of the control of muscle output itself, i.e., the physiological relationship between the lower motor neurons and effector muscles (for example see Stifani, 2014). However, uncovering the central neural circuitry underlying the planning and initiation of complex temporal sequences of movement has proven more of a challenge. Two important advancements in recent decades have allowed researchers to tackle these questions in a systematic way. The first is the capacity to measure proxy readouts of brain activity during active behavior and cognitive tasks. Here, technologies such as functional magnetic resonance imaging (fMRI), positron emission tomography (PET), and electroencephalogram (EEG) recordings have provided a wealth of data to investigate the neurophysiological underpinnings of active task engagement in health (Karuza et al., 2014; King et al., 2019) and during neurological dysfunction (Cope et al., 2021). Although these technologies remain limited in their practical application for the assessment of motor tasks (i.e., participants are generally confined to a supine or seated position and movements must be limited to reduce associated measurement artifacts; Zeng et al., 2014), their use has driven the field of cognitive neuroscience into dynamic new directions and allowed for a detailed analysis of the structure and function of movement-associated brain networks. The second major advancement has allowed researchers to gain insight into the precise neural circuits and network activity underlying movement preparation, initiation, and generation in the brain of actively behaving animals. This has arisen from the development of advanced microscopy and electrophysiological techniques for recording neural activity while simultaneously probing and manipulating circuit elements (Dombeck et al., 2007; Fois et al., 2014; Steinmetz et al., 2021). This has enabled neural activity on the network and single-cell level to be directly related to precisely timed stimulus input as well as sequences of ongoing motor behavior (e.g., Gao et al., 2018; Pakan et al., 2018a,b; Musall et al., 2019; Henschke et al., 2020, 2021; Dacre et al., 2021).

**FIGURE 1:**
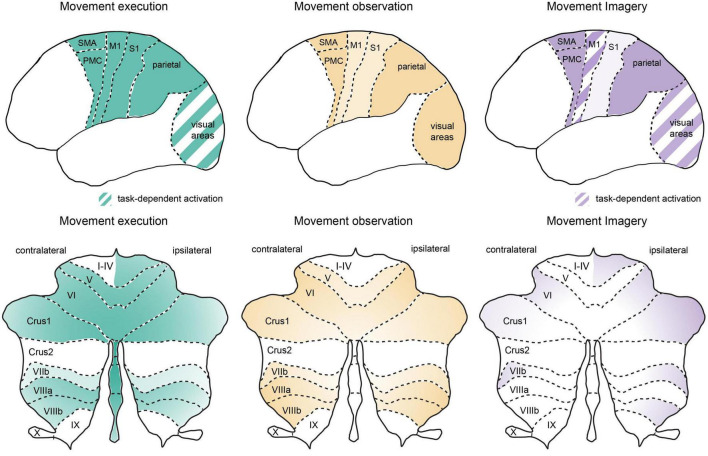
Similar global networks but different patterns of activation in cortical and cerebellar regions during movement execution, observation, and imagery. Schematic representation of cortical **(upper panel)** and cerebellar **(lower panel)** activation patterns during movement execution **(left)**, action observation **(middle)** and motor imagery **(right)**. Stronger relative activation is indicated by darker colors and striped regions indicate areas with highly task-dependent activation patterns. Overview of the activation patterns were schematized based on a summary of neuroimaging studies in humans as discussed in the text. Note that here we focus on premotor, motor, parietal, and sensory cortical networks as well as the cerebellum. An important role for lateral prefrontal cortex has been reviewed previously (see Rozzi and Fogassi, 2017) and the basal ganglia also plays an important role in cognitive-motor interactions (e.g., Yágüez et al., 1999; see also Leisman et al., 2014). M1, primary motor cortex; stocktickerPMC, pre-motor cortex; S1, primary somatosensory cortex; SMA, supplementary motor area, note SMA as indicated here also includes preSMA; visual areas, represent striate and extrastriate cortical regions, see text for details; cerebellar lobules I-X, crus 1, and crus 2 are indicated on a representation of the unfolded cerebellar cortex.

Together, these approaches have revealed that the execution of motor output and the cognitive underpinnings of motor processing both rely on distributed brain networks that span across similar cortical and subcortical regions (Figure 1). What follows is an overview of the structural and functional evidence for the activation of these distributed brain networks and their individual involvement in movement execution, observation, and imagery.

### Movement execution

When we begin a voluntary movement, the sequence of central motor planning and execution involves networks distributed across prefrontal, pre-motor, motor, and parietal cortical regions as well as subcortical structures such as the cerebellum, thalamus and basal ganglia (Haggard, 2008). Within the cerebral cortex, substantial evidence implicates medial frontal and parietal regions in movement initiation. The medial frontal regions have traditionally been associated with voluntary movements and parietal pathways with stimulus-driven action (Haggard, 2008); however, this distinction has become less clear with parietal cortex activation evident during self-initiated movements, decision making processes, and the sense of movement awareness (Cunnington et al., 2002; Sirigu et al., 2004; Haggard, 2005; Gold and Shadlen, 2007; Farrer et al., 2008; Desmurget et al., 2009). Regardless, both of these circuits converge on the primary motor cortex (M1), which then leads to movement execution through descending motor pathways to the spinal cord and ultimately the effector muscles.

During voluntary actions, fMRI studies in humans show activation in the supplementary motor area (SMA, including preSMA regions) during, and even a matter of seconds preceding, the selection of an action (Lau et al., 2004; Soon et al., 2008). These studies are supported by physiological recordings showing neural activity and neuronal recruitment in the preSMA/SMA region occurring before voluntary movements in both humans and animals (Shima and Tanji, 2000; Yazawa et al., 2000; Fried et al., 2011; Chen et al., 2017; Guo et al., 2017) and the sensation of an “urge” to perform a movement can be evoked by electrical stimulation of the SMA in humans (Fried et al., 1991). But how can we define the point of true initiation; what provides input to these pre-motor regions? The SMA receives input from lateral prefrontal regions, which are involved in planning and executing movements, particularly in the context of goal-directed behavior (Passingham et al., 2000; Borra et al., 2017; Rozzi and Fogassi, 2017). However, these SMA regions also receive input from subcortical structures such as the basal ganglia and cerebellum (Akkal et al., 2007; Sakai, 2013) and, in turn, send efferent projections back to these subcortical regions (for review see Bostan and Strick, 2018). The basal ganglia has a well-established role in suppressing unwanted movements as well as in the preparation of motor circuits for movement initiation (Schultz and Romo, 1992; for review see Simonyan, 2019), however, the importance of the connectivity between the basal ganglia and the cerebellum (Hoshi et al., 2005; Bostan and Strick, 2018) as well as the general role that the cerebellum and corticocerebellar loops play in movement initiation has been underestimated until more recently (see Apps and Watson, 2013; Thach, 2014; McAfee et al., 2022). A unique role for the cerebellum in context-dependent motor behaviors is also supported by recent optogenetics studies in rodents that perform precise circuit manipulations to demonstrate that cerebellar output directly shapes cortical activity patterns during movement initiation (Gao et al., 2018; Dacre et al., 2021; Gaffield et al., 2022). Therefore, the process of voluntary movement initiation may be more reliant on continually updated and distributed cortico-subcortical loops rather than on a single spontaneous point of initiation of “will” (Haggard, 2008; Economo et al., 2018).

For externally triggered actions, networks connecting sensory systems to parietal regions and premotor cortex (PMC) play an important role in sensory-guided movement execution. Parietal cortex has been described as the interface between sensory and motor representations (Andersen and Buneo, 2002)-with information from early sensory cortices, including both somatosensory and visual information, converging in parietal regions, which then sends projections to the PMC and M1 (Gharbawie et al., 2011). These circuits are important for sensory-guided actions such as reaching and grasping as well as sensory-guided error corrections (Grol et al., 2007; Archambault et al., 2015). Naturally, the visual input to these circuits during movement may be highly task dependent, however, specific extrastriate visual areas (e.g., V3) have been shown to be activated in monkeys during both visually guided grasping and in complete darkness (Kilintari et al., 2011). Extensive neural circuit studies in animals indicate that the interaction between various sensory and motor systems is reciprocal and can be predictive-with substantial influence of motor activity in early sensory cortices (Niell and Stryker, 2010; Pakan et al., 2016, 2018a,2018b; Leinweber et al., 2017; Ayaz et al., 2019; Henschke et al., 2021) and anticipatory responses present in somatosensory cortex even before sensory input (Umeda et al., 2019). Although caution must be taken since this activity in early sensory cortices is highly task-dependent in both animals and humans (Musall et al., 2019; Roth et al., 2020) and linked with the effects of arousal and reward (e.g., Vinck et al., 2015; Henschke et al., 2020; Roth et al., 2020). Beyond these cortico-cortical loops, the parietal-pre-motor network also has efferent connections to the cerebellum (Schmahmann and Pandya, 1989; Henschke and Pakan, 2020) and receives afferent input from the cerebellum *via* the thalamus (Clower et al., 2001; Giannetti and Molinari, 2002; Sakai, 2013; Pisano et al., 2021). These cortico-subcortical networks are activated in human fMRI studies during sensory-driven movement execution (Hardwick et al., 2018) and parietal corticocerebellar loops have been shown to be vital for the processing of dynamic motor error signals across species (Stein and Glickstein, 1992; Desmurget et al., 2001; Restuccia et al., 2007; Händel et al., 2009; Inoue and Kitazawa, 2018).

Thus, although functional overlap exists between the SMA and PMC cortical motor circuits, the parietal-pre-motor networks are thought to be especially important when immediate motor action is required, whereas lateral/medial prefrontal circuits are more involved in planning and initiating motor actions. However, in more complex real-world environments, self-initiated movements are constantly intermingled with stimulus-driven events. Given this, and the fact that both of these prefrontal/pre-motor and parietal cortical circuits have convergent output onto M1, it is appealing to take a wider systems neuroscience approach and consider the function of these brain regions together with key cortico-subcortical structures as a larger distributed brain network for motor execution (Figure 1). Things become even more complex when we consider that not all motor planning or stimulus-driven inputs results in the actual execution of a motor output. We are often passive observers of our environment, watching others perform motor actions, and yet this passive observation can lead to motor learning and subsequent improved behavioral performance (Edwards et al., 2003; Mattar and Gribble, 2005; Cross et al., 2009), particularly in combination with motor imagery (Romano-Smith et al., 2018; Marshall et al., 2020; Chye et al., 2022). Given the development of extensive brain networks for motor control, one might hypothesize that it would be anatomically and computationally efficient to simply utilize these same networks for learning through simulating motor movement, including observation and motor imagery.

### Movement observation

The initial discovery of “mirror neurons” in the ventral pre-motor cortex in monkeys, where individual cells responded to both motor execution and observation of the same action being performed (di Pellegrino et al., 1992), led to a flurry of research suggesting that the activity of these cells formed the basis for a motor representation in the brain that was fundamental to understanding motor control (Gallese et al., 1996; Rizzolatti et al., 1996; Rizzolatti and Sinigaglia, 2016). However, decades later, the precise impact of these intriguing neuronal responses remains actively debated, with the strongest consensus indicating that mirror neuron networks are involved in action imitation and low-level cognitive processing of observed actions, but others arguing for a wider role in “action understanding” (see Caligiore et al., 2013; for review see Bonini et al., 2022; Heyes and Catmur, 2022). Regardless of the wider functional implications, individual neurons that respond in some capacity to both motor execution and action observation have since been described not only in ventral pre-motor cortex but also dorsal pre-motor cortex (Tkach et al., 2007; Mazurek et al., 2018; Papadourakis and Raos, 2019), preSMA (Albertini et al., 2021), inferior parietal lobe (Fogassi et al., 2005; Bonini et al., 2010), anterior intraparietal area (Pani et al., 2014; Maeda et al., 2015; Lanzilotto et al., 2020), prefrontal cortex (area 9, Lanzilotto et al., 2017), and even the primary motor cortex (Tkach et al., 2007; Dushanova and Donoghue, 2010; Mazurek et al., 2018) in monkeys. While recordings from individual neurons in humans are understandably scarce, one study reported extracellular activity from neurons in the SMA that responded to both motor execution and action observation (Mukamel et al., 2010). Therefore, with mirror neuron properties found across this broad cortical network of sensorimotor-related regions, it is likely these neurons form a complex control system rather than acting alone in any one particular cortical area (Heyes and Catmur, 2022), similar to the function of larger motor-execution networks.

Without a specific focus on mirror neurons *per se*, a much larger body of supportive evidence comes from fMRI studies in humans that show similar networks of cortical activation occur during both motor execution and action observation (Kilner et al., 2009; Caspers et al., 2010; Molenberghs et al., 2012; Hardwick et al., 2018; and also in monkeys Raos et al., 2004, 2007), but interestingly also include the basal ganglia and cerebellum (Gazzola and Keysers, 2009; Molenberghs et al., 2012; Abdelgabar et al., 2019; Casiraghi et al., 2019; Errante and Fogassi, 2020). Although a large meta-analysis of neuroimaging studies failed to find consistent activation of subcortical regions during action observation (Caspers et al., 2010; Hardwick et al., 2018), this may be due to the bias of many neuroimaging studies to focus on cortical activation, as studies focusing on measurements within the cerebellum have consistently found significant activation in response to action observation in both humans (Van Overwalle et al., 2014; Abdelgabar et al., 2019; Casiraghi et al., 2019; Errante and Fogassi, 2020) and recently in monkeys (Raos and Savaki, 2021). Undoubtedly, various task-dependent parameters and laboratory conditions also contribute to the variability in the patterns of activation seen across studies. Interestingly, one study probing more “real-world” motor learning in trained dancers found greater activation of pre-motor, parietal, and cerebellar regions when dancers viewed moves from their own motor repertoire, in comparison to those of the opposite gender that they were familiar with viewing but not physically performing themselves (Calvo-Merino et al., 2006). This also provides evidence that the function of this action observation network is tightly linked to motor learning and motor representations in the brain and not simply visual inference alone. While the cerebellum is well-known to play a vital role in practice-dependent motor learning, it has also been suggested that these cortico-subcortical loops could specifically support the acquisition of action understanding within the mirror neuron system during observation (see Caligiore et al., 2013). Since mirror neuron properties are not innate but emerge through sensorimotor associative learning, cerebrocerebellar loops may help to coordinate cortical activity through both predictive processing (*via* cerebellar-prefrontal/pre-motor loops; Kilner et al., 2007; Guo et al., 2017; Gao et al., 2018) and establishing the temporal relationship between task-relevant events (*via* cerebellar-parietal loops; Ramnani et al., 2001; for review see D’Angelo and Casali, 2013) during both motor execution and observational learning (Caligiore et al., 2013). The importance of the cerebellum in learning through action observation is also highlighted by cerebellar lesion studies in rats, where increased performance in a spatial navigation task following strictly observational learning was abolished with cerebellar lesions (Leggio et al., 2000). Therefore, these cortico-subcortical networks play a vital role in action observation and subsequent motor learning, which should also be considered when assessing the effects of neurological lesions and neurodegeneration in humans. In fact, in Parkinson’s patients, action observation therapy has led to improvements in motor symptoms, which are associated with an increased recruitment of fronto-parietal cortical networks along with a decrease in aberrant cerebellar hyperactivity (Agosta et al., 2017), supporting a critical coordinated role for basal ganglia, cerebellar and cortical communication (Bostan and Strick, 2018; Errante and Fogassi, 2020).

**FIGURE 2:**
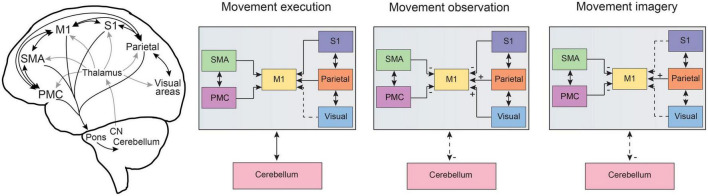
Information flow across common distributed brain networks during movement execution, observation and imagery. Schematic representations of the distributed brain networks (left) show the gross anatomical cortico-cortical and corticocerebellar connections for brain regions related to motor processing. The general comparison of information flow for cortico-cortical as well as cortico-cerebellar pathways is summarized for movement execution, observation, and imagery. Dashed lines represent altered connectivity across conditions and ± symbols indicate changes in input across regions as based on a summary of neuroimaging studies and neurophysiological recordings in humans and monkeys as discussed in the text. Note that here we focus on pre-motor, motor, parietal, and sensory cortical networks as well as corticocerebellar pathways. An important role for lateral prefrontal cortex has been reviewed previously (see Rozzi and Fogassi, 2017) and the basal ganglia also plays an important role in cognitive-motor interactions (e.g., Yágüez et al., 1999; see also Leisman et al., 2014). CN, cerebellar nuclei; M1, primary motor cortex; PMC, pre-motor cortex; S1, primary somatosensory cortex; SMA, supplementary motor area, note SMA also includes preSMA; visual, refers to visual cortical pathways including striate and extrastriate regions, see text for details.

Therefore, there is substantial evidence that the same distributed brain networks are activated during both motor execution and action observation. This begs the question of why the activation of these motor systems during action observation does not result in overt motor behavior. In this regard, it is important to consider differences between the subnetworks that are involved and key changes in the activation balance within these distributed brain networks (Figure 2). For instance, in the study by Mukamel et al. (2010), electrophysiological recordings in the human SMA demonstrated heterogeneous responses on the single-cell level, with some neurons showing excitation following both execution and observation but a subset of neurons responding with excitation to execution and inhibition during observation. Similar findings have been reported from M1 neuronal recordings in monkeys (Dushanova and Donoghue, 2010; Vigneswaran et al., 2013; Mazurek et al., 2018), indicating that the final output of M1 to spinal circuitry is reduced in magnitude during action observation and may not be sufficient to produce overt muscle activity. Indeed, this dissociation of motor movements from M1 activity has been exploited in the control of brain computer interface (BCI) devices (Schieber, 2011; Chaudhary et al., 2022), although some have suggested that posterior parietal regions may be even more effective placements for BCI control in this regard (Aflalo et al., 2015). In neuroimaging studies, the balance of activation has also been reported to be stronger in pre-motor regions during observation and stronger in S1 and M1 during motor execution (Raos et al., 2004, 2007; Gazzola and Keysers, 2009), and differences in the spatial pattern of activity between execution and observation were also reported in anterior parietal regions (Dinstein et al., 2008). Finally, the cerebellum shows significant shared regional activation during executed and observed actions, including lobules VI, VIIb, VIIIa and to a lesser degree V, crus I, crus II and VIIIb (Abdelgabar et al., 2019; King et al., 2019; Errante and Fogassi, 2020; Raos and Savaki, 2021), although these regions do not follow strict lobule boundaries but are congruent with more recent frameworks for functional cerebellar organization (King et al., 2019; Guell and Schmahmann, 2020). However, there is also a shift in activation to more lateral aspects of the cerebellum during action observation in comparison to action execution (King et al., 2019; Raos and Savaki, 2021). This is congruent with a generalized mediolateral functional gradient within the cerebellum, with a motor, visuomotor, and cognitive focus extending from medial to lateral regions, respectively (Guell et al., 2018; D’Mello et al., 2020). With the precise parasagittal oriented modular organization of the cerebellum (Apps and Hawkes, 2009), shifts along this mediolateral functional gradient during movement execution and observation could enable the rapid transition between cortical execution and observational states through coordinated communication between the cerebellum and cerebral cortex (Likova et al., 2021; McAfee et al., 2022).

### Movement imagery

Motor imagery has been proposed to evoke a neural simulation network that would shape the motor system in anticipation for motor execution and ultimately provide information on the feasibility of potential actions and strengthen motor learning (Jeannerod, 2001; Ladda et al., 2021). As such, it is not surprising that motor imagery has also been proposed as a cognitive tool that could facilitate training and rehabilitation (for review see Mulder, 2007; Yoxon and Welsh, 2020). However, this approach alone has produced varying levels of success (Chye et al., 2022), likely in part due to a lack of standardization in the use of motor imagery (although see Moreno-Verdú et al., 2022) and the systematic study of motor imagery presenting a number of challenges due to its covert and introspective nature. In this regard, the advancement of neuroimaging techniques has hastened progress to uncover the neurophysiological bases of motor imagery in humans, providing the capability to examine activated brain networks underlying these covert cognitive processes. Studies using motor imagery have consistently report activation in brain regions that are also involved in movement execution, including parietal regions, pre-motor cortices, SMA and the cerebellum (see Figure 1; Lotze et al., 1999; Grèzes and Decety, 2000; Munzert et al., 2009; Hétu et al., 2013; Hardwick et al., 2018). However, within these common cortical networks, the neural representation of motor imagery vs. motor execution is also separable using multivariate approaches (Sharma and Baron, 2013; Zabicki et al., 2017). Notably the level of activation in sensory cortical regions, S1 and visual cortex, are substantially decreased during motor imagery in comparison to both motor execution and action observation (Hardwick et al., 2018). Additionally, the activation of visual cortical areas (both striate and extrastriate) can be highly task-dependent in motor imagery (Knauff et al., 2000; Kosslyn and Thompson, 2003), depending on if the subject is asked to use kinesthetic vs. visual imagery (Solodkin et al., 2004; Guillot et al., 2009). In the cerebellum specifically, motor imagery again shifts cerebellar activation to more lateral positions in the cerebellar hemispheres, even in comparison to action observation (Lotze et al., 1999; Hardwick et al., 2018; King et al., 2019). Overall levels of cerebellar activation are also lower in comparison to motor execution (Lotze et al., 1999; Hardwick et al., 2018), which may stem from a lack of ascending somatosensory input through spinocerebellar pathways. However, during motor imagery, the levels of cerebellar activation are also lower in comparison to action observation (which also lacks ascending somatosensory input), suggesting that the additional reduction in cortical output from sensory cortices (S1 and visual areas) may reduce cerebellar activation further *via* altered activity in cerebrocerebellar loops (Figure 2), as these sensory cortices are also highly connected to the cerebellum (Sultan et al., 2012; Henschke and Pakan, 2020; Xue et al., 2021).

Similar to the case of action observation, the involvement of the motor cortex itself has led to the most controversy, with the majority of neuroimaging studies not reporting consistent M1 activation during motor imagery (Hétu et al., 2013; Hardwick et al., 2018). Methodological factors such as specific task-dependent criteria, fMRI/PET sensitivity, imagery modality, and task instructions may all play a role in the discrepancy between studies (for discussion see Munzert et al., 2009; Hétu et al., 2013; Hardwick et al., 2018). However, transcranial magnetic stimulation (TMS) studies have found that motor imagery can increase the excitability of M1 (Munzert et al., 2009; Loporto et al., 2011). This may indicate that similar to during action observation, responses in M1 may also involve more subtle changes in excitatory/inhibitory balance on the neuronal level that are not easy to resolve with functional neuroimaging methodology and associated temporal resolution. Indeed, a study measuring electrocorticographic cortical activity reported a 25% reduction in M1 activity during motor imagery compared to actual movement (Miller et al., 2010), which is consistent with results using dynamic causal modeling with high temporal resolution fMRI data that found a strong suppressive influence of SMA on M1 during motor imagery (Kasess et al., 2008).

While convincing evidence for a role of M1 during action observation has come from the extensive investigation of mirror neurons and other direct neuronal recordings in animals, the investigation of motor imagery presents unique challenges in this regard. Due to the covert nature of mental imagery, it has been exceedingly difficult to carry out studies of these phenomena using animal models. One proposed path includes an adapted definition of a more perceptual-based “mental imagery” as the ability to maintain an active representation of sensory or perceptual details in the absence of actual sensory input (Blaisdell, 2019). In this way, neuronal activity could be assessed in relation to working memory tasks, probing associative and causal learning, or through inferences about unobservable outcomes (Waldmann et al., 2012; Fast et al., 2016; Blaisdell, 2019). However, it is possible that the capacity for mental imagery is uniquely human (Dennett, 1995; Shettleworth, 2010), and animals may be more likely to experience perceptual events without sensory input in the form of expectation or memory. While the questions of mental imagery in animals currently appears largely intractable to convincing empirical validation, a phenomenon in humans referred to as *aphantasia*, i.e., the inability to engage in mental imagery (Dance et al., 2022), provides an interesting opportunity to discover more about how mental imagery contributes to our motor and perceptual processing on the neurophysiological level. This field is still in its infancy, but some early studies suggest that motor simulations may indeed be impaired in individuals with aphantasia (Dupont et al., 2022), who also present with measurable alterations in behavioral and neural signatures during mental imagery (Milton et al., 2021; Dupont et al., 2022).

## Discussion

The execution of motor movements is carried out by a distributed parietal-frontal network and key subcortical structures including the cerebellum. These same networks support functions related to more abstract motor cognition including action observation and motor imagery. Here we have provided an overview of the wealth of neuroimaging data in humans, as well as key supportive evidence from animal models, that has enabled a detailed comparison between the common network elements and the subnetwork specializations across these various forms of motor-related processing.

A growing awareness of the function of the cerebellum outside the strict domain of motor coordination has resulted in an increased focus on this brain structure in recent neuroimaging studies. While this is a welcome addition to the field, there are some caveats to consider when evaluating the results of these neuroimaging studies. Beyond the traditional issues of interpreting small sample sizes, measuring individual differences across neuroimaging data, and complex considerations of statistical analyses (Brown and Behrmann, 2017; Gordon et al., 2017; Specht, 2020), one must also be cautious when drawing conclusions about cerebellar structure and function from early fMRI studies. Historically, many studies assessing “whole brain” activation using neuroimaging techniques are focused on the neocortex and often exclude the cerebellum entirely from data analysis and reporting. This is partly due to particular challenges for mapping functional neuroimaging in the cerebellum that are only recently starting to be addressed (Diedrichsen et al., 2010; Schlerf et al., 2014). For instance, the small size and functional heterogeneity of the cerebellum requires additional consideration for normalization and alignment methods in comparison to the neocortex (e.g., Makris et al., 2005). Recent neuroimaging studies dedicated to functional specializations in the cerebellum have contributed to a wider appreciation of the diverse roles of the cerebellum and importance of cerebrocerebellum communication (Buckner et al., 2011; Guell et al., 2018; Ji et al., 2019; King et al., 2019; Guell and Schmahmann, 2020; Xue et al., 2021).

Further advances in neuroimaging techniques will continue to help researchers address the fascinating question of how the brain implements flexible distributed networks for motor control that are simultaneously utilized and adapted by covert cognitive and perceptual processes. For instance, through a series of shared distributed brain networks, a dancer is able to observe their teacher performing a choreographed routine, mentally rehearse it, and then seamlessly produce the precise motor control needed for flawless execution of a complex series of movements. While we may not all possess the skills of a trained dancer, this seamless transition from sensory input–to motor cognition–to motor execution is constantly ongoing in our normal daily lives. Further, understanding this inherent plasticity within motor systems may help to improve the available repertoire of tools for neurological rehabilitation following motor dysfunction.

## Author contributions

Both authors contributed to the design and conceptualization, literature search, and writing of the review and approved the final submitted version.
